# Supplementary material for: Comprehensive mapping of adaptation of the avian influenza polymerase protein PB2 to humans
Source: eLife. 2019 Apr 30;8:e45079. doi: 10.7554/eLife.45079 (PMC6491042; doi:10.7554/eLife.45079)
Supplement: Supplementary file 3. [file elife-45079-supp3.zip › FileS2_JupyterNotebooks/Fig5_MapDiffSelToStructure.html]

Fig5\_MapDiffSelToStructure


# Map differential selection to structures¶

We generated pymol scripts to display positive differential selection values on various PB2 structures.

| Structure | pdb name | Input txt files | Output pymol scripts |
| --- | --- | --- | --- |
| Polymerase in transcription pre-initiation form | 4wsb | preamble   setview   colorfullstructure   topA549 | map\_fullstructure.py   map\_topA549.py   map\_positive\_diff\_seq.py |
| Polymerase in apo form | 59d8 | preamble   setview   colorfullstructure   topA549 | map\_fullstructure.py   map\_topA549.py   map\_positive\_diff\_seq.py |
| PB2 in complex with RNA pol II | 6f5o | preamble   setview   MartinResidues   topA549 | map\_MartinResidues.py   map\_positive\_diff\_seq.py |
| PB2 in complex with importin | 4uae | preamble   setview   PumroyResidues   topA549 | map\_PumroyResidues.py   map\_positive\_diff\_seq.py |

Import modules, define directories

In [1]:

```
import os
import shutil
import pandas as pd
import numpy as np
from colour import Color

import dms_tools2
import dms_tools2.dssp

import matplotlib as mpl
import matplotlib.pyplot as plt
import seaborn as sns
sns.set_style("whitegrid")
sns.set_context("talk", font_scale=1.3)
```

In [2]:

```
## Set plotting parameters/preferences
# Set matplotlib rcParams for figures
import seaborn as sns
sns.set(context='paper', style='ticks', palette='deep', font='Arial', font_scale=1.042, color_codes=True,
        rc = {'font.size': 10,
 'axes.labelsize': 10,
 'axes.titlesize': 10,
 'xtick.labelsize': 9,
 'ytick.labelsize': 9,
 'legend.fontsize': 9,
 'axes.linewidth': 1.0,
 'grid.linewidth': 0.8,
 'lines.linewidth': 1,
 'lines.markersize': 4.5,
 'patch.linewidth': 0.8,
 'xtick.major.width': 1.0,
 'ytick.major.width': 1.0,
 'xtick.minor.width': 0.8,
 'ytick.minor.width': 0.8,
 'xtick.major.size': 4.5,
 'ytick.major.size': 4.5,
 'xtick.minor.size': 3,
 'ytick.minor.size': 3}
       )
palette ={"None":"gray",
          "A549":"#d95f02",
         }
# sns.plotting_context()
```

Generate `topA549.txt`

In [3]:

```
# Gather prior annotations for known human/mammalian adaptive mutations
mutsAdaptive = pd.read_table('data/Muts_HumanAvian.txt')
knownAdaptive = (set(mutsAdaptive[mutsAdaptive['ExptVerified']=='Yes']['Site']))
# Gather top adaptive mutations in A549
dmssummarydf = pd.read_csv('results/diffsel/summary_prefs_effects_diffsel.csv')
topA549 = (set(dmssummarydf[dmssummarydf['Experimentally adaptive in']=='A549']['site']))
# Intersect
knownAdaptiveAndTopA549 = (knownAdaptive & topA549)

# Set colors for each set of mutations
mutsets = [((knownAdaptive, 'knownAdaptive'), 'blue'), 
           ((topA549, 'topA549'), 'red'), 
           ((knownAdaptiveAndTopA549, 'knownAdaptiveAndTopA549'), 'magenta')]

# Write to file
outfile = 'pymol/topA549.txt'
f = open(outfile, 'w')
f.write('metric = \'topA549\'\n')
f.write('cmd.color(\'white\', \'S009\')\n\n')
for ((mutlist, mutlistname), col) in mutsets:
    f.write('# {0}\n'.format(mutlistname))
    f.write('for r in [{0}]:\n'.format(', '.join([str(mut) for mut in mutlist])))
    f.write('\tcmd.color(\'{0}\', \'resi {{0}} and S009\'.format(r))\n'.format(col))
    f.write('\tcmd.show(\'spheres\', \'resi {{0}} and S009\'.format(r))\n\n')
f.close()
```

Generate pymol scripts

In [4]:

```
# Standard across all structures
postamble = 'pymol/postamble.txt'
fullstructure = 'pymol/colorfullstructure.txt'
topA549 = 'pymol/topA549.txt'
pumroy = 'pymol/PumroyResidues.txt'
martin = 'pymol/MartinResidues.txt'
specialmetricfiles = {'fullstructure': fullstructure,
                     'topA549': topA549, 
                     'pumroy': pumroy,
                     'martin': martin}
# For non-specialmetrics
diffsel_file = 'results/diffsel/summary_A549vCCL141-meansitediffsel.csv'
metricdf = pd.read_csv(diffsel_file).sort_values('site')
colspectrum = 'white_red'

def write_pymol_script(structure, metric, metricdf, colspectrum):
    outfile = 'pymol/{0}_map_{1}.py'.format(structure, metric)
    preamble = 'pymol/preamble_{0}.txt'.format(structure)
    setview = 'pymol/setviewamble_{0}.txt'.format(structure)

    f = open(outfile, 'w')

    with open(preamble, 'r') as addtext:
        for line in addtext.readlines():
            f.write(line)
            if metric=='fullstructure' and line=='### To get PB2 only\n': 
                #don't print lines that will remove non-PB2 subunits
                break
        f.write('\n\n')
    
    # Write commands to color structure
    if metric in specialmetricfiles:
        with open(specialmetricfiles[metric], 'r') as addtext:
            for line in addtext.readlines():
                f.write(line)
            f.write('\n\n')
    else:
        f.write("metric = \'{0}\'\n".format(metric))
        for row in metricdf.itertuples(index=True, name='Pandas'):
            site, met = (getattr(row, "site"), getattr(row, metric))
            f.write("cmd.alter(\'resi {0}\', \'b = {1}\')\n".format(site, met))
        f.write('\n')
        f.write("cmd.spectrum(\'b\', \'{0}\', \'S009\')\n".format(colspectrum))
        f.write('\n')

    with open(setview, 'r') as addtext:
        for line in addtext.readlines():
            f.write(line)
    f.write('\n')
    with open(postamble, 'r') as addtext:
        for line in addtext.readlines():
            f.write(line)

    f.close()
```

In [5]:

```
structures = ['4wsb', '5d98']
metrics = ['fullstructure', 'topA549', 'positive_diffsel']

for structure in structures:
    for metric in metrics:
        write_pymol_script(structure, metric, metricdf, colspectrum)
```

In [6]:

```
structures = ['6f5o']
metrics = ['martin', 'positive_diffsel', 'topA549']

for structure in structures:
    for metric in metrics:
        write_pymol_script(structure, metric, metricdf, colspectrum)
```

In [7]:

```
structures = ['4uad']
metrics = ['pumroy', 'positive_diffsel', 'topA549']

for structure in structures:
    for metric in metrics:
        write_pymol_script(structure, metric, metricdf, colspectrum)
```

## Are differentially selected residues on the surface of PB2/polymerase?¶

Calculate RSA for top A549 differentially selected residues.

In [8]:

```
# Manually generate new pdb files for S009-PB2 superimposed on 4wsb and 5d98 structures, using following scripts.
# S009-PB2 is saved as chain X.
# For each structure, I made two files: one with only PB2, and one with PB2 as part of polymerase complex.
!ls pymol/*superS009.py
```

```
pymol/4wsb_superS009.py  pymol/5d98_superS009.py
```

In [9]:

```
# Convert the following files to dssp: https://swift.cmbi.umcn.nl/gv/dssp/
!ls pymol/*superS009*pdb
```

```
pymol/4wsb_superS009.pdb	   pymol/5d98_superS009.pdb
pymol/4wsb_superS009_S009only.pdb  pymol/5d98_superS009_S009only.pdb
```

In [10]:

```
# Converted to dssp
!ls pymol/*superS009*dssp
```

```
pymol/4wsb_superS009.dssp	    pymol/5d98_superS009.dssp
pymol/4wsb_superS009_S009only.dssp  pymol/5d98_superS009_S009only.dssp
```

In [11]:

```
dssplist = []
structures = ['4wsb', '5d98']
contexts = [('full pol', ''), ('S009 only', '_S009only')]
for structure in structures:
    for context, contextlab in contexts:
        dsspdf = dms_tools2.dssp.processDSSP('pymol/{0}_superS009{1}.dssp'.format(structure, contextlab), chain='X')
        dsspdf['structure'] = structure
        dsspdf['context'] = context
        dssplist.append(dsspdf)
dssps = pd.concat(dssplist)
print(dssps.head())
# Gather annotations of mutations (known, novel)
annot = pd.read_csv('results/diffsel/summary_prefs_effects_diffsel.csv')[['site', 'Experimentally adaptive in']]
annot = annot[annot['Experimentally adaptive in']=='A549'].drop_duplicates()
print(annot.head())

dssps = pd.merge(left=dssps, right=annot, on='site', how='left')
dssps.fillna('None', inplace=True)
```

```
   site amino_acid  ASA       RSA SS SS_class structure   context
0     1          M  104  0.464286  -     loop      4wsb  full pol
1     2          E  155  0.695067  H    helix      4wsb  full pol
2     3          R   61  0.222628  H    helix      4wsb  full pol
3     4          I    3  0.015228  H    helix      4wsb  full pol
4     5          K   77  0.326271  H    helix      4wsb  full pol
      site Experimentally adaptive in
921    163                       A549
1385   627                       A549
1459   701                       A549
1700   183                       A549
2049   532                       A549
```

In [12]:

```
dssps[dssps['site']==1]
```

Out[12]:

|  | site | amino\_acid | ASA | RSA | SS | SS\_class | structure | context | Experimentally adaptive in |
| --- | --- | --- | --- | --- | --- | --- | --- | --- | --- |
| 0 | 1 | M | 104 | 0.464286 | - | loop | 4wsb | full pol | None |
| 733 | 1 | M | 162 | 0.723214 | - | loop | 4wsb | S009 only | None |
| 1466 | 1 | M | 95 | 0.424107 | - | loop | 5d98 | full pol | None |
| 2221 | 1 | M | 157 | 0.700893 | - | loop | 5d98 | S009 only | None |

In [13]:

```
dssp_4wsb = pd.merge(left=dssps[(dssps['structure']=='4wsb') & (dssps['context']=='full pol')][['site', 'amino_acid', 'Experimentally adaptive in', 'RSA']],
                    right=dssps[(dssps['structure']=='4wsb') & (dssps['context']=='S009 only')][['site', 'amino_acid', 'Experimentally adaptive in', 'RSA']],
                    on=['site', 'amino_acid', 'Experimentally adaptive in'],
                    suffixes=['_4wsbfullpol','_4wsbS009only']
                   )

dssp_5d98 = pd.merge(left=dssps[(dssps['structure']=='5d98') & (dssps['context']=='full pol')][['site', 'amino_acid', 'Experimentally adaptive in', 'RSA']],
                    right=dssps[(dssps['structure']=='5d98') & (dssps['context']=='S009 only')][['site', 'amino_acid', 'Experimentally adaptive in', 'RSA']],
                    on=['site', 'amino_acid', 'Experimentally adaptive in'],
                    suffixes=['_5d98fullpol','_5d98S009only']
                   )

dsspwide = pd.merge(left=dssp_4wsb, right=dssp_5d98,on=['site', 'amino_acid', 'Experimentally adaptive in'])
dsspwide.head()
```

Out[13]:

|  | site | amino\_acid | Experimentally adaptive in | RSA\_4wsbfullpol | RSA\_4wsbS009only | RSA\_5d98fullpol | RSA\_5d98S009only |
| --- | --- | --- | --- | --- | --- | --- | --- |
| 0 | 1 | M | None | 0.464286 | 0.723214 | 0.424107 | 0.700893 |
| 1 | 2 | E | None | 0.695067 | 0.695067 | 0.663677 | 0.663677 |
| 2 | 3 | R | None | 0.222628 | 0.773723 | 0.445255 | 0.791971 |
| 3 | 4 | I | None | 0.015228 | 0.512690 | 0.035533 | 0.614213 |
| 4 | 5 | K | None | 0.326271 | 0.457627 | 0.309322 | 0.347458 |

dssp\_bystructure = pd.merge(left=dssps[dssps['context']=='full pol'][['structure', 'site', 'amino\_acid', 'Experimentally adaptive in', 'RSA']],
right=dssps[dssps['context']=='S009 only'][['structure', 'site', 'amino\_acid', 'Experimentally adaptive in', 'RSA']],
on=['structure', 'site', 'amino\_acid', 'Experimentally adaptive in'],
suffixes=['\_fullpol','\_S009only']
)
dssp\_bystructure.head()sns.set\_style("ticks")
df = dsspwide
hue = 'Experimentally adaptive in'
x, xlab = 'RSA\_4wsbfullpol', '4wsb full pol'
y, ylab = 'RSA\_5d98fullpol', '5d98 full pol'
hue\_order = ['None', 'A549']
hue\_kws = dict(alpha=[0.2,1], facecolors=[None,None], s=[20,20], linewidths=[1,1], marker=["o","o"])
lowlim, highlim = -0.05, 1.05
g = sns.FacetGrid(df, height=3, aspect=1,
hue=hue, hue\_order=hue\_order, hue\_kws=hue\_kws,
palette=palette,
despine=False,
)
g = (g.map(plt.scatter, x, y)
.set\_axis\_labels(xlab, ylab)
.set(xlim=(lowlim, highlim), ylim=(lowlim, highlim),
xticks=[0, 0.2, 0.4, 0.6, 0.8, 1], yticks=[0, 0.2, 0.4, 0.6, 0.8, 1])
)
plt.plot([0.2, 0.2], [lowlim, highlim], linewidth=1, color='b', alpha=0.5)
plt.plot([lowlim, highlim], [0.2, 0.2], linewidth=1, color='b', alpha=0.5)
plt.plot([lowlim, highlim], [lowlim, highlim], linewidth=1, color='b', alpha=0.5)
resi\_xy = zip(df[df[hue]=='A549'][x], df[df[hue]=='A549'][y])
resi\_sites = df[df[hue]=='A549']['site']
residues = zip(resi\_sites, resi\_xy)
for (resi\_site, (resi\_x, resi\_y)) in residues:
plt.text(resi\_x+0.02, resi\_y, resi\_site, horizontalalignment='left', verticalalignment='bottom')

In [14]:

```
def scatterplotRSA(df, hue, x, xlab, y, ylab):
    hue_order = ['None', 'A549']
    hue_kws = dict(alpha=[0.2,1], facecolors=[None,None], s=[20,20], linewidths=[1,1], marker=["o","o"])
    lowlim, highlim = -0.05, 1.05
    g = sns.FacetGrid(df, height=3, aspect=1,
                      hue=hue, hue_order=hue_order, hue_kws=hue_kws,
                      palette=palette, 
                      despine=False,
                     )
    g = (g.map(plt.scatter, x, y)
         .set_axis_labels(xlab, ylab)
         .set(xlim=(lowlim, highlim), ylim=(lowlim, highlim), 
             xticks=[0, 0.2, 0.4, 0.6, 0.8, 1], yticks=[0, 0.2, 0.4, 0.6, 0.8, 1])
        )
    plt.plot([0.2, 0.2], [lowlim, highlim], linewidth=1, color='b', alpha=0.5)
    plt.plot([lowlim, highlim], [0.2, 0.2], linewidth=1, color='b', alpha=0.5)
    plt.plot([lowlim, highlim], [lowlim, highlim], linewidth=1, color='b', alpha=0.5)

    resi_xy = zip(df[df[hue]=='A549'][x], df[df[hue]=='A549'][y])
    resi_sites = df[df[hue]=='A549']['site']
    residues = zip(resi_sites, resi_xy)
    for (resi_site, (resi_x, resi_y)) in residues:
        plt.text(resi_x+0.02, resi_y, resi_site, horizontalalignment='left', verticalalignment='bottom')
    plt.savefig('pymol/{0}_{1}.pdf'.format(x, y), dpi=300, bbox_inches='tight')
```

In [15]:

```
sns.set_style("ticks")
df = dsspwide
hue = 'Experimentally adaptive in'
x, xlab = 'RSA_4wsbfullpol', '4wsb full pol'
y, ylab = 'RSA_5d98fullpol', '5d98 full pol'
scatterplotRSA(df, hue, x, xlab, y, ylab)
```

In [16]:

```
x, xlab = 'RSA_4wsbfullpol', '4wsb full pol'
y, ylab = 'RSA_4wsbS009only', '4wsb PB2 only'
scatterplotRSA(df, hue, x, xlab, y, ylab)
```

In [17]:

```
x, xlab = 'RSA_5d98fullpol', '5d98 full pol'
y, ylab = 'RSA_5d98S009only', '5d98 PB2 only'
scatterplotRSA(df, hue, x, xlab, y, ylab)
```

In [18]:

```
dsspwide[dsspwide['Experimentally adaptive in']=='A549']
```

Out[18]:

|  | site | amino\_acid | Experimentally adaptive in | RSA\_4wsbfullpol | RSA\_4wsbS009only | RSA\_5d98fullpol | RSA\_5d98S009only |
| --- | --- | --- | --- | --- | --- | --- | --- |
| 8 | 9 | D | A549 | 0.590674 | 0.590674 | 0.450777 | 0.450777 |
| 68 | 69 | E | A549 | 0.399103 | 0.399103 | 0.300448 | 0.300448 |
| 81 | 82 | N | A549 | 0.374359 | 0.476923 | 0.400000 | 0.400000 |
| 155 | 156 | A | A549 | 0.534884 | 0.534884 | 0.627907 | 0.627907 |
| 157 | 158 | E | A549 | 0.201794 | 0.201794 | 0.103139 | 0.103139 |
| 162 | 163 | I | A549 | 0.000000 | 0.060914 | 0.040609 | 0.086294 |
| 168 | 169 | P | A549 | 0.327044 | 0.471698 | 0.201258 | 0.201258 |
| 175 | 176 | I | A549 | 0.659898 | 0.746193 | 0.279188 | 0.416244 |
| 181 | 182 | Q | A549 | 0.004444 | 0.004444 | 0.106667 | 0.106667 |
| 182 | 183 | L | A549 | 0.258706 | 0.258706 | 0.064677 | 0.064677 |
| 189 | 190 | K | A549 | 0.588983 | 0.588983 | 0.033898 | 0.033898 |
| 291 | 292 | I | A549 | 0.441624 | 0.441624 | 0.187817 | 0.187817 |
| 354 | 355 | R | A549 | 0.587591 | 0.587591 | 0.419708 | 0.419708 |
| 512 | 521 | T | A549 | 0.418605 | 0.418605 | 0.604651 | 0.604651 |
| 513 | 522 | Q | A549 | 0.168889 | 0.168889 | 0.577778 | 0.577778 |
| 523 | 532 | S | A549 | 0.541935 | 0.541935 | 0.458065 | 0.458065 |
| 618 | 627 | E | A549 | 0.403587 | 0.403587 | 0.573991 | 0.573991 |
| 660 | 669 | G | A549 | 0.288462 | 0.625000 | 0.336538 | 0.336538 |
| 667 | 676 | T | A549 | 0.261628 | 0.261628 | 0.069767 | 0.069767 |
| 675 | 684 | A | A549 | 0.891473 | 0.891473 | 0.449612 | 0.449612 |
| 689 | 698 | G | A549 | 0.384615 | 0.384615 | 0.009615 | 0.211538 |
| 692 | 701 | D | A549 | 0.409326 | 0.409326 | 0.253886 | 0.419689 |

## Copy files to paper figures directory¶

In [19]:

```
paperdir = './paper'
figuresdir = os.path.join(paperdir, 'figures/')
myfiguresdir = os.path.join(figuresdir, 'Fig5/')
if not os.path.isdir(myfiguresdir):
    os.mkdir(myfiguresdir)

filespy = !ls pymol/*.py
filespdb = !ls pymol/*.pdb
filespdf = !ls pymol/*.pdf
for f in filespy + filespdb + filespdf:
    shutil.copy(f, myfiguresdir)
```

In [20]:

```
filespy + filespdb + filespdf
```

Out[20]:

```
['pymol/4uad_map_positive_diffsel.py',
 'pymol/4uad_map_pumroy.py',
 'pymol/4uad_map_topA549.py',
 'pymol/4wsb_map_fullstructure.py',
 'pymol/4wsb_map_positive_diffsel.py',
 'pymol/4wsb_map_topA549.py',
 'pymol/4wsb_superS009.py',
 'pymol/5d98_map_fullstructure.py',
 'pymol/5d98_map_positive_diffsel.py',
 'pymol/5d98_map_topA549.py',
 'pymol/5d98_superS009.py',
 'pymol/6f5o_map_martin.py',
 'pymol/6f5o_map_positive_diffsel.py',
 'pymol/6f5o_map_topA549.py',
 'pymol/4wsb_superS009.pdb',
 'pymol/4wsb_superS009_S009only.pdb',
 'pymol/5d98_superS009.pdb',
 'pymol/5d98_superS009_S009only.pdb',
 'pymol/S009PB2_4uae.pdb',
 'pymol/S009PB2_4wsb.pdb',
 'pymol/S009PB2_5d98.pdb',
 'pymol/S009PB2_6f5o.pdb',
 'pymol/RSA_4wsbfullpol_RSA_4wsbS009only.pdf',
 'pymol/RSA_4wsbfullpol_RSA_5d98fullpol.pdf',
 'pymol/RSA_5d98fullpol_RSA_5d98S009only.pdf']
```
